# Synthesis, Structure and Biological Activity of CIA and CIB, Two α-Conotoxins from the Predation-Evoked Venom of *Conus catus*

**DOI:** 10.3390/toxins10060222

**Published:** 2018-06-01

**Authors:** Julien Giribaldi, David Wilson, Annette Nicke, Yamina El Hamdaoui, Guillaume Laconde, Adèle Faucherre, Hamid Moha Ou Maati, Norelle L. Daly, Christine Enjalbal, Sébastien Dutertre

**Affiliations:** 1 Institut des Biomolécules Max Mousseron, UMR 5247, Université de Montpellier—CNRS, 34095 Montpellier, France; julien.giribaldi@umontpellier.fr (J.G.); guillaume.laconde@umontpellier.fr (G.L.); christine.enjalbal@umontpellier.fr (C.E.); 2 Centre for Biodiscovery and Molecular Development of Therapeutics, Australian Institute of Tropical Health and Medicine, James Cook University, Cairns, QLD 4878, Australia; david.wilson4@jcu.edu.au (D.W.); norelle.daly@jcu.edu.au (N.L.D.); 3 Walther Straub Institute of Pharmacology and Toxicology, Faculty of Medicine, LMU Munich, Nußbaumstraße 26, 80336 Munich, Germany; annette.nicke@lrz.uni-muenchen.de (A.N.); elhamdao@uni-mainz.de (Y.E.H.); 4 Département de Physiologie, Institut de Génomique Fonctionnelle, CNRS/INSERM UMR 5203, Université de Montpellier, 34095 Montpellier, France; Adele.Faucherre@igf.cnrs.fr (A.F.); Hamid.Moha@igf.cnrs.fr (H.M.O.M.)

**Keywords:** conotoxins, Conus catus, electrophysiology, in vivo, nicotinic receptors, structure, synthesis

## Abstract

Cone snails produce a fast-acting and often paralyzing venom that is usually injected into their prey or predator through a hypodermic needle-like modified radula tooth. Many diverse compounds are found in their venom including small molecules, peptides and enzymes. However, peptidic toxins called conotoxins (10–40 residues and 2–4 disulfide bonds) largely dominate these cocktails. These disulfide rich toxins are very valuable pharmacological tools for investigating the function of ions channels, G-protein coupled receptors, transporters and enzymes. Here, we report on the synthesis, structure determination and biological activities of two α-conotoxins, CIA and CIB, found in the predatory venom of the piscivorous species *Conus catus*. CIA is a typical 3/5 α-conotoxin that blocks the rat muscle type nAChR with an IC_50_ of 5.7 nM. Interestingly, CIA also inhibits the neuronal rat nAChR subtype α3β2 with an IC_50_ of 2.06 μM. CIB is a 4/7 α-conotoxin that blocks rat neuronal nAChR subtypes, including α3β2 (IC_50_ = 128.9 nM) and α7 (IC_50_ = 1.51 μM). High resolution NMR structures revealed typical α-conotoxin folds for both peptides. We also investigated the in vivo effects of these toxins on fish, since both peptides were identified in the predatory venom of *C. catus*. Consistent with their pharmacology, CIA was highly paralytic to zebrafish (ED_50_ = 110 μg/kg), whereas CIB did not affect the mobility of the fish. In conclusion, CIA likely participates in prey capture through muscle paralysis, while the putative ecological role of CIB remains to be elucidated.

## 1. Introduction

Cone snails are predatory marine mollusks comprising more than 800 different species [1]. They capture prey using a venom gland that produces a fast-acting paralyzing venom injected through a hypodermic needle-like radula tooth [2]. Conotoxins, small (10–40 residues) and highly constrained peptides (2 to 4 disulfide bridges), are the main components of cone snail venom, which also contains small molecules and enzymes [3]. Conotoxins are classified into various gene superfamilies based on their conserved signal sequence. Further classification is based on their cysteine framework and their target receptor [4,5]. Conotoxins often have highly specific and selective biological activity, and many of them proved to be very valuable pharmacological tools or even drug leads [6,7,8]. Indeed their high selectivity and affinity combined with their small size make these toxins good candidates for the design of therapeutic peptides or peptide mimetics [9,10]. One particular class of conotoxins, the α-conotoxins, acts as antagonists of the nicotinic acetylcholine receptors (nAChRs), a diverse family of ligand-gated ion channels formed by the pentameric assembly of homologous subunits [8].

Both neuronal and muscle type nAChR associated channels open in response to binding of the neurotransmitter acetylcholine [11], and antagonism of nAChR may be of interest for the treatment of pain, nicotine addiction or epilepsy [12,13], whereas agonistic action is desired for treating neurological disorders. The numerous neuronal nAChRs subtypes are involved in a wide variety of biological mechanisms [14,15,16,17] in the central nervous system [14,15] but also in the peripheral nervous system [16] and the immune system [17]. In the central nervous system, they are involved in neurological disorders such as Parkinson’s disease, Alzheimer’s disease, epilepsy and schizophrenia [18,19], but also in more complex mechanisms such as learning, memory or mood control [20]. Whereas some neuronal subtypes are already validated targets, several combinations of neuronal nAChR subunits remain orphan of selective ligands and therefore more efforts need to be directed at finding or designing novel α-conotoxins with tailored pharmacological profiles.

Historically, α-conotoxins have proved to be remarkable probes for characterizing nAChRs subtypes and establishing their physiological/pathophysiological roles [15,21]. The first α-conotoxins to be characterized were isolated from *Conus geographus* venom gland extracts [22]. Their potent paralytic action at the neuromuscular junction prompted the interpretation of their ecological role as being essential for prey capture [23]. However, recent findings demonstrated that in the case of *C. geographus*, α-conotoxins are injected massively for defense purposes, not for prey capture [2,24]. In order to determine the ecological role of α-conotoxins in other fish-hunting species, we report on the synthesis, structure determination and biological activities of two new α-conotoxins CIA and CIB discovered in the venom gland transcriptome and confirmed by proteomic analysis to be present in the injected predatory venom of the piscivorous species *Conus catus* [25]. Both CIA and CIB were investigated at the functional and structural level, as well as tested for in vivo effect on fish. 

## 2. Results

### 2.1. Chemical Synthesis

**Figure 1:**
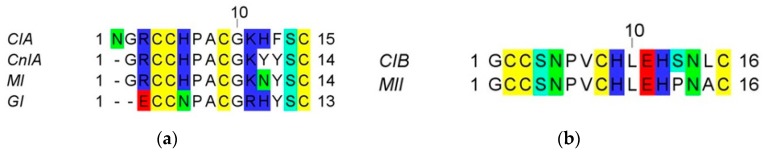
Sequences of α-conotoxins. (**a**) Alignment of CIA α-conotoxin with other closely related 3/5 α-conotoxins; (**b**) Alignment of 4/7 α-conotoxin CIB with MII.

Two α-conotoxins, CIA and CIB (Figure 1), were found to be relatively abundant in the predatory venom of the piscivorous species *Conus catus* [25]. To determine the biological activity, structure and ecological role of these conotoxins, both peptides were manually synthesized using Fmoc-based solid-phase peptide synthesis (see experimental procedures). Assuming the canonical globular disulfide bond connectivity for α-conotoxins (C_1_–C_3_; C_2_–C_4_), linear peptides were folded using a regioselective protection (Acm group) of cysteine residues C_1_–C_3_. According to the number of residues within the loops, CIA and CIB are 3/5 and 4/7 α-conotoxins and we expected that they would target muscle type and neuronal type nAChRs, respectively [26,27,28,29].

**Figure 2:**
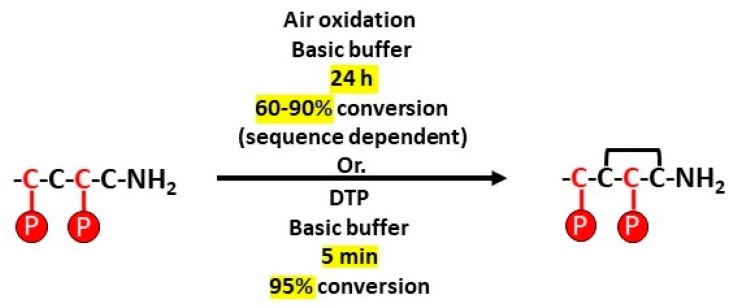
Comparison of disulfide bond formation between free cysteine residues by using air oxidation or DTP. P stands for usual cysteine lateral chain protective groups.

As part of this two-step folding strategy, the standard procedure for the formation of the first disulfide bond from the two non-protected cysteine residues (C_2_–C_4_) typically consists of stirring the linear peptide in an aqueous basic buffer [30]. However, when the kinetics of disulfide bond formation are too slow, it is common to add potassium hexacyanoferrate or glutathione and oxidized glutathione [31] to facilitate the bridge formation [32,33]. Formation of the first disulfide bridge in an aqueous basic buffer (without any additives) took 24 h to obtain 60% conversion (based on UV chromatogram) and 40 h to obtain 50% conversion for CIA and CIB respectively. Alternatively, we found that DTP (2,2′-Dithiopyridine) was very effective in greatly accelerating the disulfide bridge formation. Surprisingly, this reagent is not widely used, despite its effectiveness being demonstrated by Maruyama et al. [34] 20 years ago. We used this method on several other conotoxins and to our experience, if used in a diluted medium, an excess of DTP induces the quasi-total formation of the intramolecular disulfide bridge in less than 10 min (Figure 2). The second disulfide bond is formed between C_1_-C_3_ after removal of the Acm protecting groups.

**Figure 3:**
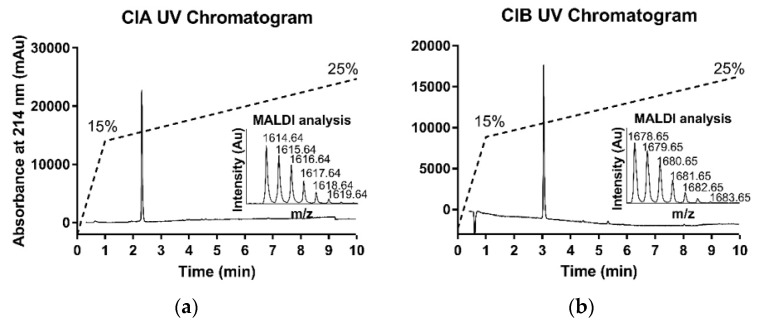
HPLC and MS analyses of synthetic CIA and CIB. (**a**) Synthetic folded CIA UV chromatogram at 214 nm and mass spectrometry MALDI analysis; (**b**) Synthetic folded CIB UV chromatogram at 214 nm and mass spectrometry MALDI analysis. Dashed line indicates the acetonitrile gradient.

The homogeneity of folded CIA and CIB was assessed by analytical RP-HPLC and MS (Figure 3). MALDI-MS(+) confirmed a monoisotopic mass of 1614.63 Da (calculated 1614.64 Da for [M + H]^+^) for CIA and 1678.65 Da (calculated 1678.64 Da for [M + H]^+^) for CIB. Overall, 7.7 mg and 3.4 mg of pure CIA and CIB were obtained, respectively.

### 2.2. Electrophysiology

**Figure 4:**
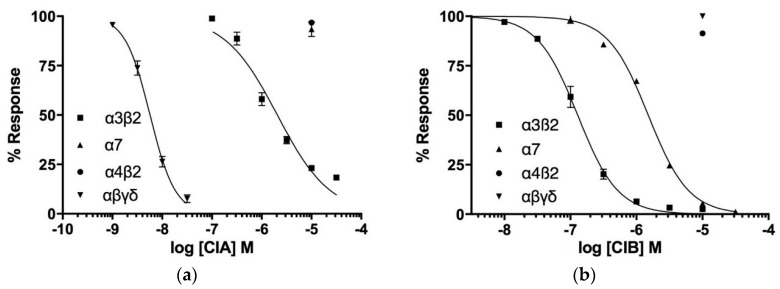
Concentration-response analysis of α-conotoxins CIA (**a**) and CIB (**b**) on wild type nAChRs. The indicated subunit combinations were expressed in *Xenopus laevis* oocytes and analyzed by 2-electrode voltage clamp at -70 mV. Responses to 2-s pulses of 100 µM ACh (or nicotine in case of the α7 receptor) were recorded after a 3-min preincubation with the indicated toxin. Each point represents the mean of measurements from at least 3 different oocytes. Error bars represent S.E.M.

Next, the biological activity of CIA and CIB was investigated using a two-electrode voltage clamp analysis on three neuronal nAChRs subtypes (α3β2, α7, α4β2) and the muscle type (α1)_2_δγβ1 nAChR from rat expressed in *Xenopus laevis* oocytes. As expected, the 3/5 α-conotoxin CIA potently blocks the muscle type nAChR with an IC_50_ of 5.7 nM (Figure 4a). Surprisingly, CIA also inhibits the neuronal nAChR subtype α3β2, although with >350-fold lower affinity (IC_50_ of 2.06 μM), whereas no activity was detected on the two other subtypes at concentration up to 10 μM. In contrast, CIB is a 4/7 α-conotoxin that blocks the neuronal nAChR α3β2 subtype with an IC_50_ of 128.9 nM and α7 subtype with an IC_50_ of 1.51 μM (Figure 4b). No activity was detected on the muscle and α4β2 subtypes at concentrations up to 10 μM.

### 2.3. NMR Spectroscopy

**Figure 5:**
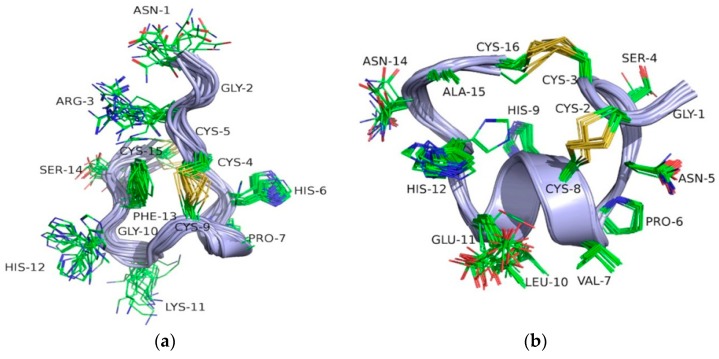
Three-dimensional structures of CIA and CIB. The 20 lowest energy NMR structures, superimposed over the backbone atoms for CIA (**a**) and CIB (**b**). The backbone is shown in ribbon format and the side-chains in stick format.

High resolution NMR spectroscopy allowed us to determine the three-dimensional structures of both CIA and CIB (Figure 5). The structure statistics are given in Table 1. The NMR spectra for CIA indicate the presence of multiple conformations based on the presence of additional cross-peaks, whereas CIB indicates the presence of a single conformation. The structure determined for CIA was for the major conformation, as the minor conformation displayed weak peaks. The calculated structures are well-defined with low RMSDs for residues 5–11. However, CIB has a larger number of NOE restraints and consequently a better defined structure. CIA has a 3_10_ helix from residues 7–9 and CIB an α-helix from residues 6 to 11. CIA has a similar three-dimensional structure to the closely related peptide, CnIA. Both peptides have a 3_10_ helix involving the Pro7-Ala8-Cys10 sequence (CIA numbering). The ensembles of CIA and CnIA (PDB code 1B45) superimpose with an RMSD of 1.26 Å (calculated using MOLMOL). Similarly, CIB is structurally very similar to MII, where both peptides have a central α-helical region comprising residues 6–11 in CIB and residues 7–11 in MII. The ensembles of CIB and MII (PDB code 1MII) superimpose with an RMSD of 0.55 Å (calculated using MOLMOL).

The conformational heterogeneity observed for CIA is common to most α-3/5 conotoxins. Indeed, Favreau et al. reported similar conformational heterogeneity in solution for α-3/5 conotoxin CnIA, but this also holds true for α-3/5 GI and α-3/5 MI [26,35,36]. This heterogeneity could be due to cis to trans isomerization of a peptidic bond, but at this stage there is no data to support this hypothesis [26]. The presence of a minor conformation has been identified by Maslennikov et al. for α-conotoxin GI [35]. These two conformations are interconvertible in solution and differ in the regions of the second loop and C terminus [35]. Therefore, it is not possible to conclude whether only one or both of the two conformations is responsible for the activity, or whether each conformation has the same level of activity.

### 2.4. In vivo Bioassays

**Figure 6:**
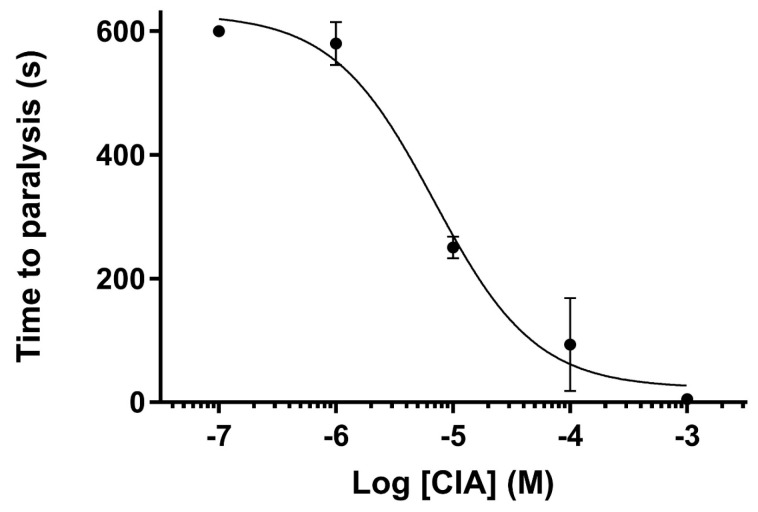
Paralytic effect of CIA on zebrafish. Paralysis induced by conotoxin α-CIA shows a dose-dependent effect, with an IC_50_ of 6.88 μM.

Since both CIA and CIB were detected in the predatory venom of the piscivorous *C. catus*, they were injected into fish (*Danio rerio*) in order to infer the possible ecological role of each conotoxin in prey capture. Intramuscular injection of α-conotoxin CIA produces a rapid flaccid paralysis of skeletal muscles, as evidenced by a loss of equilibrium of the fish, and ultimately a complete immobilization. Paralysis induced by conotoxin α-CIA was a dose-dependent effect, with an IC_50_ of 6.88 μM (Figure 6). Based on the calculated average weight of our adult zebrafish (0.5 g) and the volume injected (5 μL), CIA has an ED_50_ of 110 μg/kg. Injection of up to 1 mM of CIB, however, does not produce any noticeable effect on the locomotion of zebrafish, which is consistent with the absence of activity on muscle nAChR.

## 3. Discussion

Animal venoms are generally complex mixtures of hundreds or more biologically active compounds. It is assumed that each of these molecules has been selected through evolution for a specific ecological role. Whereas pharmacologically characterized conotoxins from cone snail venom duct extracts have traditionally been associated to primary roles in prey-capture due to their paralytic action, recent findings suggest other possible venom-ecology relationships [2]. Indeed, independently collected predation and defense-evoked venoms unexpectedly showed that the potent paralytic conotoxins well characterized from *C. geographus* were almost exclusively injected to defend against predators, not for prey capture. This is contrary to the consensus literature published on this topic for the past 30 years. Noteworthy, piscivorous cones of the Gastridium clade such as *C. geographus* and *C. tulipa* employ a rather unusual and unique prey capture strategy to catch fish, producing an apparent sedative effect through passive release of venom components in the surrounding water [2]. On the other hand, species in the largest clade of fish-hunting cones, namely the Pionoconus, use a “Taser-like” effect to rapidly stun fish. This effect is thought to be the result of a combination of two synergic conotoxin types: δ-conotoxins, which inhibit the inactivation of voltage-gated sodium channels, and κ-conotoxins, which block voltage-dependent potassium channels [37]. However, the predation-evoked venom of *C. striatus* and *C. consors*, two of the largest species of Pionoconus, shows a composition completely devoid of these conotoxins, composed instead of nearly exclusively unrelated κA-conotoxins, and the occasional α- and ω-conotoxins [38,39]. Similarly in the predation-evoked venom of one of the smallest fish-hunting species, *C. catus*, Himaya et al. reported mainly the presence of κA-conotoxins, but consistently also some vertebrate-active and paralytic α-, ω-, and μ-conotoxins in significant amounts [25].

Based on this previous study [25], the sequences corresponding to two major α-conotoxins with the cysteine pattern CCX_3_CX_5_C and CCX_4_CX_7_C were synthesized and their biological activity assessed using electrophysiology and fish bioassays. The first α-conotoxin, named CIA, is a typical muscle type α-3/5 conotoxin and its sequence closely resembles those of other α-3/5 conotoxins such as MI, GI and CnIA. CIA blocks the muscle type nAChR with high affinity (~5 nM), but surprisingly, it was found to also block the α3β2 neuronal subtype of nAChR, albeit with lower potency (~2 μM). To our knowledge, CIA is the only known nAChR muscle type α-conotoxin that can also target the neuronal α3β2 subtype with significant affinity. Whereas the molecular basis for this neuronal nAChR activity remains to be investigated, conserved proline and tyrosine/phenylalanine residues (corresponding to position 7 and 13 in CIA sequence, respectively) have been shown to be crucial for the strong hydrophobic interaction between the δ subunit of the muscle type nAChR and conotoxin MI, suggesting a similar mode of action [27,40] for CIA.

**Figure 7:**
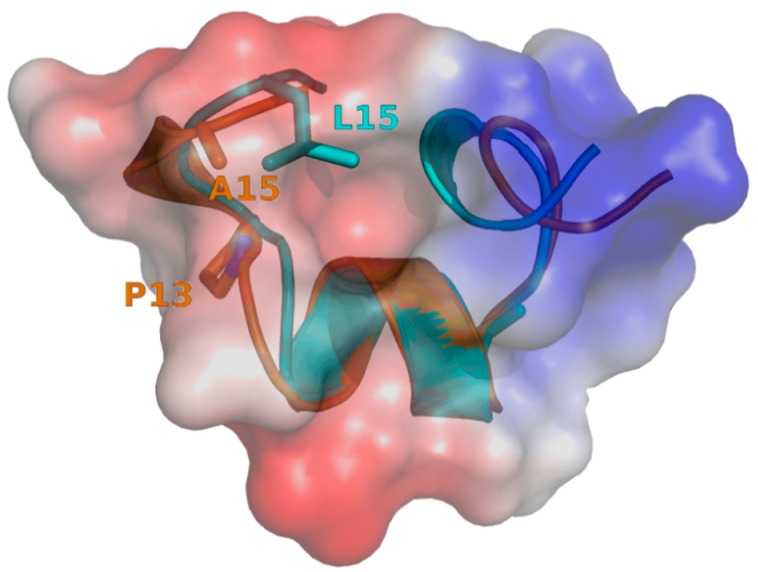
Charge distribution of CIB (orange) and MII (blue; PDB: 1MII), superimposed over residues 1 to 16. Charges are represented with different shades from red (negative charge states) to blue (positive charge states). A cavity appears in the molecular surface of CIB, which is partially filled by L15 in MII.

The second α-conotoxin, CIB, blocks the neuronal nAChR α3β2 subtype with an IC_50_ of 128.9 nM and the α7 subtype with an IC_50_ of 1.51 μM. The sequence of CIB is most similar to α-conotoxin MII, sharing 14 out of 16 amino acids. However, its potency towards α3β2 is approximatively 29-fold lower compared to MII [41]. The two different residues that most likely account for this difference are proline in position 13 for CIB instead of a serine for MII and alanine in position 15 for CIB instead of leucine for MII. By comparing the three-dimensional structures, it appears that the proline residue in position 13 of CIB induces a kink that prevents the alanine in position 15 from filling the hydrophobic pocket in the same way the leucine does in MII (Figure 7). However, Dutertre et al. [42] using an AChBP model showed that MII possesses an hydrophobic core (Pro-6, Val-7 and Leu 10) where Pro-6 is involved in a direct interaction with β2-Leu-119 and additional hydrophobic contacts due to interactions of Val-7 and Leu-10 with β2-Val-109 and β2-Phe-117 respectively. Docking studies would be very helpful to further investigate how this difference at position 15, which seems to not be directly involved in the binding with the receptor, can explain the significant loss of activity of CIB compared to MII.

Conotoxins are traditionally tested on mammalian systems, including human and rodent receptors. However, in the case of these two α-conotoxins CIA and CIB found in the predatory venom of *C. catus*, it was of interest to evaluate their activity on their natural prey (here fish for piscivorous cone snails). Intramuscular injection of α-conotoxin CIA into fish causes flaccid paralysis of the skeletal muscles with an effective dose ED_50_ = 110 μg/kg, highlighting a potent biological effect compatible with a role in prey capture. At the highest dose (1 mM) tested, the paralysis is almost instantaneous, however this dose is unlikely to be biologically relevant. Indeed, it was demonstrated for *Conus purpurascens* that most conotoxins expressed in injected venoms range up to 3.51–121.01 μM within a single sting sample [43]. Therefore, the quasi-instantaneous tetanic paralysis provoked by *Conus catus* sting [44] is likely due to the most abundant conotoxins injected, namely the κA-conotoxins [25,45]. Indeed, Kelley et al. observed that the injection of purified κA-conotoxins is able to reproduce the biological effects of the whole injected venom on fish prey [45]. The role of α-3/5 conotoxins in prey envenomation is most likely secondary, and the resulting blockade of the neuromuscular junction may prevent the escape response of a fish prey that would recover from the “Taser-effect” of κA-conotoxins. Interestingly α-3/5 conotoxin CIA is also found in *Conus achatinus* and *Conus consors* [31,46], belonging also to the Pionoconus clade. On the other hand, injection of α-conotoxin CIB into fish did not cause any significant change compared to the control. The role of conotoxins targeting neuronal nAChRs in prey capture remains to be elucidated, but we propose that they may interfere with sensory circuitry and the escape response of prey.

## 4. Materials and Methods

### 4.1. Abbreviations

Acm, acetamidomethyl; ACN, acetonitrile; Boc, *tert*-butoxycarbonyle; CHCA, α-Cyano-4-hydroxycinnamic acid; DCM, Dichloromethane; DIEA, diisopropylethylamine; DMF, *N*,*N*′-dimethylformamide; DTP, 2,2′-Dithiopyridine; eq, equivalent; ESI-MS, electrospray ionization mass spectrometry; Fmoc, fluorenylmethoxycarbonyl; HATU, 1 [*Bis*(dimethylamino)methylene]-1*H*-1,2,3-triazolo[4,5-*b*]pyridinium 3-oxid hexafluorophosphate; LC/MS, liquid chromatography/mass spectrometry; MALDI, Matrix Assisted Laser Desorption/Ionization; MeOH, methanol; nAChR, nicotinic acetylcholine receptor; NMR, Nuclear Magnetic Resonance; Pbf, pentamethyl-dihydrobenzofuran-5-sulfonyl; RP-HPLC, reversed phase high performance liquid chromatography; SPPS, solid phase peptide synthesis; t-Bu, *tert*-butyl; TFA, trifluoroacetic acid; TIS, triisopropylsilane; Tris, 2-Amino-2-(hydroxymethyl)propane-1,3-diol; Trt, trityl; UV, ultra-violet.

### 4.2. Chemical Synthesis

DMF, DIEA, ACN, TIS, TFA, piperidine and all others reagents were obtained from Sigma-Aldrich (Saint-Louis, MI, USA) or Merck (Darmstadt, Allemagne) and were used as supplied. Fmoc (L) amino acid derivatives and HATU were purchased from Iris Biotech (Marktredwitz, Germany). AmphiSpheres 40 RAM resin (0.37 mmol/g 75–150 µm) was purchased from Agilent Technologies (Les Ulis, France). The side chain protecting groups for amino acids are t-Bu for Asp, Glu, Ser, Thr and Tyr; Trt for Cys_3,16_ of CIB and Cys_5,15_ of CIA, Acm for Cys_2,8_ of CIB and Cys_4,9_ of CIA; Trt for Gln; Pbf for Arg; Boc for Lys and Trp. CIA and CIB were manually synthesized by using the Fmoc-based solid-phase peptide synthesis technique on a VWR (Radnor, PA, USA) microplate shaker. All Fmoc amino acids and HATU were dissolved in DMF to reach 0.5 M. Chain elongation was performed step by step using 0.1 mmol of AmphiSpheres 40 RAM resin. Fmoc deprotection was performed with 20% piperidine in DMF two times, each for 1 min at room temperature, then the resin is washed three times with DMF. Each Fmoc-protected amino acid (5 eq) was coupled in the presence of HATU (5 eq) and *N*,*N*-diisopropylethylamine (DIEA, 10 eq) in DMF at room temperature for two min. After completion of coupling reaction, the resin was sequentially washed two times with DMF. Cleavage of peptide from the resin and removal of side-chain protecting groups were carried out using 10 mL of a solution containing TFA/TIS/H_2_O (95:2.5:2.5, *v*/*v*/*v*) for 15 min at 60 °C. After the resin was removed by filtration and washed three times with dichloromethane. Dichloromethane and TFA are removed under vacuum then cold diethyl ether was added to precipitate the peptide. Crude peptides were purified by preparative RP-HPLC, and their purity were confirmed by LC/ESI-MS. A twostep oxidation procedure was then carried out. Trt groups were removed during the cleavage step while the Acm protective groups are resistant to cleavage conditions. The first disulfide bridge is formed from free cysteines with the use of DTP and the second disulfide bridge is formed by concomitant deprotection and oxidation of the Acm groups [30,34].

### 4.3. Mass Spectrometry

Solvents used for LC/MS were of HPLC grade. The LC/MS system consisted of a Waters (Milford, OH, USA) Alliance 2695 HPLC, coupled to a Waters Micromass ZQ spectrometer (electrospray ionization mode, ESI+). All the analyses were carried out using a Chromolith (Fontenay sous Bois, France) HighResolution RP-18e (4.6 × 25 mm, 15 nm–1.15 μm particle size, flow rate 3.0 mL/min) column. A flow rate of 3 mL/min and a gradient of 0–100% B over 2.5 min were used. Eluent A: water/0.1% HCO_2_H; eluent B: acetonitrile/0.1% HCO_2_H. UV detection was performed at 214 nm. Electrospray mass spectra were acquired at a solvent flow rate of 200 µL/min. Nitrogen was used for both the nebulizing and drying gas. The data were obtained in a scan mode ranging from 100 to 1000 *m*/*z* or 250 to 1500 *m*/*z* to in 0.7 s intervals. MALDI mass spectrometry analyses were performed on an Ultraflex III instrument from Bruker Daltonics (Champs sur Marne, France). Each sample was analyzed from CHCA matrix deposit in positive-ion mode.

### 4.4. Preparative RP-HPLC

Preparative RP-HPLC was run on a Gilson PLC 2250 Purification system (Villiers le Bel, France) instrument using a preparative column (Waters DeltaPak C18 Radial-Pak Cartridge, 100 Å, 40 × 100 mm, 15 μm particle size, flow rate 50.0 mL/min). Buffer A was 0.1% TFA in water, and buffer B was 0.1% TFA in acetonitrile.

### 4.5. Electrophysiology Measurements

cDNAs of rat nAChR subunits used in this study were provided by J. Patrick (Baylor College of Medicine, Houston, TX, USA) and subcloned into the oocyte expression vector pNKS2. cRNA was synthesized with the SP6 mMessage mMachine Kit (Ambion, Austin, TX, USA) and adjusted to a concentration of 0.5 mg/mL. nAChR subunit RNAs were mixed in the ratios 1:1 (α3/β2), 5:1 (α4/β2), and 2:1:1:1 (α1/β1/δ/γ). *Xenopus laevis* (Nasco International, Fort Atkinson, WI, USA) oocytes were injected with 50 nl aliquots of cRNA (Nanoject automatic oocyte injector, Drummond Scientific, Broomall, PA). Antagonist dose-response curves were measured as described previously (Dutertre et al., 2005) in ND96 (96 mM NaCl, 2 mM KCl, 1 mM CaCl_2_, 1 mM MgCl_2_, and 5 mM HEPES at pH 7.4). In brief, current responses to acetylcholine were measured 1–6 days after cRNA injection and recorded at −70 mV using a Turbo Tec 05X Amplifier (NPI Electronic, Tamm, Germany) and Cell Works software. A standard concentration of 100 μM ACh for α3β2, α4β2 nAChRs and (α1)_2_δγβ muscle nAChR and a standard concentration of 100 µM nicotine for the α7 nAChR was used to keep the data comparable to previous studies. A fast and reproducible solution exchange (<300 ms) was achieved with a 50-μL funnel-shaped oocyte chamber combined with a fast vertical solution flow fed through a custom-made manifold mounted immediately above the oocyte. Agonist pulses were applied for 2 s at 4-min intervals. Following one minute of perfusion directly after the agonist application, peptides were applied in a static bath for 3 min. IC_50_ values were calculated from a nonlinear fit of the Hill equation to the data (Prism version 4.0; GraphPad Software, Inc., San Diego, CA). Data are presented as means ± S.E.M. from at least three oocytes.

### 4.6. NMR Spectroscopy

Lyophilized synthetic peptides were resuspended to a final concentration of ~2.5 mM in 90%H_2_O:10%D_2_O. 2D ^1^H-^1^H TOCSY, ^1^H-^1^H NOESY, ^1^H-^1^H DQF-COSY, ^1^H-^15^N HSQC, and ^1^H-^13^C HSQC spectra were acquired at 290 K using a 600 MHz AVANCE III NMR spectrometer (Bruker, Karlsruhe, Germany) equipped with a cryogenically cooled probe. All spectra were recorded with an interscan delay of 1 s. NOESY spectra were acquired with mixing times of 200–250 ms, and TOCSY spectra were acquired with isotropic mixing periods of 80 ms. Two-dimensional spectra were collected over 4096 data points in the f2 dimension and 512 increments in the f1 dimension over a spectral width of 12 ppm. Standard Bruker pulse sequences were used with an excitation sculpting scheme for solvent suppression. Slowly exchanging amide protons were detected by acquiring a series of one-dimensional and TOCSY spectra over a 24 h period, following dissolution of the peptides in D_2_O. The two-dimensional NOESY spectra of CIA and CIB were automatically assigned and an ensemble of structures calculated using the program CYANA [47]. Torsion-angle restraints from TALOS+ were used in the structure calculations. Distance restraints between the beta carbons and sulfur atoms of the cysteine residues that are disulfide bonded were included in the structure calculations. Restraints were included between Cys I-Cys II and Cys II-Cys IV. The final structures were visualized using Pymol.

### 4.7. In vivo Bioassay

Zebrafish were maintained under standardized conditions and experiments were conducted in accordance with the European Communities council directive 2010/63. Briefly, CIA and CIB were diluted in milli-Q water and 5 µL of incremental doses were injected intramuscularly into adult zebrafish with a 10 µL Neuros Syringe from Hamilton (Bonaduz, Switzerland). Each dose was repeated three times on three different fishes to determine error bars. The onset of paralysis was measured over a maximum observation time of 10 min. Paralysis was considered total when the fish went on its back. We performed negative control experiments according to the same protocol by injecting milli-Q water instead of toxins.

## Figures and Tables

**Table 1:** Structural statistics for CIA and CIB.

	CIA	CIB
**Experimental restraints**		
Interproton distance restraints	69	98
*Intraresidue*	30	30
*Sequential*	28	47
*Medium range* (*i-j < 5*)	11	18
*Long range* (*i-j ≥5*)	0	3
Disulfide-bond restraints	4	4
Dihedral-angle restraints	21	22
**R.m.s deviations from mean coordinate structure (Å)**		
Backbone atoms	0.95 ± 0.33	0.48 ± 0.16
Backbone atoms (res 5–11)	0.08 ± 0.04	0.19 ± 0.10
All heavy atoms	1.92 ± 0.46	0.95 ± 0.22
All heavy atoms (res 5–11)	0.39 ± 0.41	0.80 ± 0.19
**Ramachandran Statistics**		
Clashscore, all atoms	0 ± 0	0 ± 0
% in most favoured region	85.7 ± 0	91 ± 10
MolProbity score	2.13 ± 0.14	1.68 ± 0.45
